# A Cascade Flexible Neural Forest Model for Cancer Subtypes Classification on Gene Expression Data

**DOI:** 10.1155/2021/6480456

**Published:** 2021-10-05

**Authors:** Lianxin Zhong, Qingfang Meng, Yuehui Chen

**Affiliations:** School of Information Science and Engineering, University of Jinan, Jinan 250022, China

## Abstract

The correct classification of cancer subtypes is of great significance for the in-depth study of cancer pathogenesis and the realization of accurate treatment for cancer patients. In recent years, the classification of cancer subtypes using deep neural networks and gene expression data has become a hot topic. However, most classifiers may face the challenges of overfitting and low classification accuracy when dealing with small sample size and high-dimensional biological data. In this paper, the Cascade Flexible Neural Forest (CFNForest) Model was proposed to accomplish cancer subtype classification. CFNForest extended the traditional flexible neural tree structure to FNT Group Forest exploiting a bagging ensemble strategy and could automatically generate the model's structure and parameters. In order to deepen the FNT Group Forest without introducing new hyperparameters, the multilayer cascade framework was exploited to design the FNT Group Forest model, which transformed features between levels and improved the performance of the model. The proposed CFNForest model also improved the operational efficiency and the robustness of the model by sample selection mechanism between layers and setting different weights for the output of each layer. To accomplish cancer subtype classification, FNT Group Forest with different feature sets was used to enrich the structural diversity of the model, which make it more suitable for processing small sample size datasets. The experiments on RNA-seq gene expression data showed that CFNForest effectively improves the accuracy of cancer subtype classification. The classification results have good robustness.

## 1. Introduction

Cancer is a heterogeneous lesion caused by the loss of the normal regulation of local tissue cell growth at the gene level under the action of carcinogenic factors [1]. Nowadays, cancer has become one of the major causes of human death [2]. Traditional cancer research methods were mostly based on clinical experience. Doctors made diagnoses by analyzing clinical data and referring to a limited number of cases. However, the molecular expression level of cancer is highly heterogeneous, which means that there are many molecular subtypes in cancer tissue. Cancer patients with the same symptoms can show significant prognostic differences under the same treatment regimens [3]. Heterogeneity is one of the fundamental features of cancer, and it is also the biggest challenge for the development of precision therapy for cancer [4].

The process of occurrence, development, and metastasis of cancer is very complex. Findings have shown that different cancer subtypes differ significantly in multiple gene expression data [5]. Therefore, cancer research at the genetic level is of great importance for cancer treatment and diagnosis. The advent of Next-Generation Sequencing has led to an explosive growth in the amount of gene expression data. However, making the best use of these data to make accurate predictions for different cancer subtypes is a serious challenge for researchers [6].

Although all the genes in the tissue cells were recorded in the gene expression profile of the sample, only few genes were associated with the classification of cancer subtypes [7]. In addition, the data structure of gene expression is complex, the information redundancy is very high, and the correlation between genes is very strong. Traditional biological research methods are difficult to deal with gene expression data effectively [8]. Therefore, researchers are in urgent need of new and targeted analysis methods.

Deep learning, a branch of artificial intelligence, has become a powerful tool for biological data analysis and processing through the use of backpropagation, hierarchical processing, and various optimization algorithms [5]. Gene expression data is characterized by high dimensionality, high redundancy, and extreme spatial complexity. After processing the gene expression data with machine learning method, it can not only identify the relationship between genes effectively, but also establish more accurate prediction models. This provides an important technical support for the correct classification of cancer subtypes and is of great significance for both theoretical research and practical clinical applications of cancer [9].

In recent years, many researchers have made various attempts to use deep learning methods to study cancer subtype classification. Fakoor et al. [10] used unsupervised feature learning on a deep stack autoencoder for processing gene expression data and completed cancer detection and cancer subtype analysis using it. The method improved the accuracy of cancer subtype classification. A scalable method to process gene expression data for different cancer subtypes is also provided in the article. Khademi and Nedialkov [11] proposed a probabilistic graphical model (PGM) to predict and diagnose breast cancer. The model addresses the problem of poor learning due to problems such as high and small size of genomic datasets through diverse learning and deep belief networks. In the article, the authors used SoftMax nodes to integrate PGMs and DBNs constructed from clinical and microarray data, and the approach showed good results in cancer subtype classification, cancer recurrence, and metastasis prediction tasks on real datasets. Guo et al. [12] proposed a deep learning framework called BCDForest. The traditional deep forest model was improved by drawing on the advantages of deep learning. Experimental results show that the model can exhibit better classification performance when classifying subtypes of various cancer. Karabulut and Ibrikci [13] proposed a discriminative deep belief network (DDBN) model for cancer subtype classification. In this paper, the problem of high dimensionality and imbalance of gene expression data was solved. Experimental results showed that the proposed model outperformed other classifiers in the accuracy of cancer subtype classification. Xiao et al. [14] proposed a deep learning based multimodal ensemble approach for cancer prediction using deep learning methods to fuse multiple machine learning classification models. Experimental results on RNA-seq datasets of lung, gastric, and breast cancers showed that the method can effectively improve the accuracy of cancer subtype classification.

In this paper, the Cascade Flexible Neural Forest (CFNForest) was proposed for cancer subtype classification. This model is a deep neural network ensemble framework based on FNT Group Forest. Compared with traditional deep neural network models, CFNForest can automatically optimize the structure and parameters of the internal neural network during the training process. Compared with deep models built on nondifferentiable models, the proposed model can stratify genetic features without discrete input features. CFNForest integrated multiple classifier ensemble strategies and improves the overall classification accuracy of the model through feature conversion between levels. Through the introduction of enhanced sample and feature optimization mechanism, the model still showed good classification performance on small sample size datasets. Experimental results showed that CFNForest consistently outperforms the state-of-the-art methods in the classification of breast invasive carcinoma, glioblastoma multiforme, and lung cancer using RNA-seq gene expression data.

## 2. Materials and Methods

### 2.1. Datasets

The RNA sequence gene expression data were used in this paper, which were downloaded from The Cancer Genome Atlas (TCGA) [15]. Three types of cancers were downloaded from the TCGA database and sorted: Breast Invasive Carcinoma (BRCA), Glioblastoma Multiforme (GBM), and Lung Cancer (LUNG). The labeling of each sample is based on real clinical data of cancer patients provided by TCGA. There are four basic subtypes of BRCA: Basal-like (98/∼19.06%), HER2-enriched (58/∼11.28%), Luminal-A (231/∼44.94%), and Luminal-B (127/∼24.72%). A total of 514 valid data samples were available. There are three subtypes in LUNG: Bronchioid (104/∼37.82%), Magnoid (72/∼26.18%), and Squamoid (99/∼36.0), with 275 effective samples. In GBM there are four basic subtypes: Classical (42/∼25.62%), Mesenchymal (55/∼33.5%), Neural (28/∼17.1%), and Proneural (39/∼23.78%), and the number of valid samples available is 164. The details of three cancer types are shown in Table 1.

The preprocessing steps are outlier deletion, missing data imputation, and normalization: if the gene expression data has more than 20% missing value in a patient, the patient data will be filtered; for the missing data, *K*-nearest neighbor is used to fill in; the normalization of cancer gene expression data is processed as follows:

(1) $$\begin{array}{c} \tilde{f}=\frac{f-E\left(f\right)}{\sqrt{\text{Var}\left(f\right)}}, \end{array}$$

where *f* is the gene expression data feature and $\tilde{f}$ is the normalized gene expression feature. *E*(*f*) and Var(*f*) are the mean and variance of *f*.

### 2.2. The Flexible Neural Tree Model

The advantages of deep neural networks, such as layer-by-layer processing, backpropagation, and sufficient model complexity, make them show strong performance advantages in several fields of machine learning. However, when setting the structure of neural networks, there are almost countless combinations of layers of neural networks, numbers of neurons, and links between neurons [16]. The quality of the model often depended on the experience of the researcher. When the network structure is too complex, not only is the model prone to the risk of overfitting, but the complex training process also brings extremely high computing costs [17].

When the network structure is too simple, the classification and prediction performance of the model will not be very good. Based on this, Chen et al. proposed the flexible neural tree (FNT) model [16–18]. The automatic optimization of the structure and the automatic optimization of the parameters are the two most important parts in the process of constructing a FNT model. In this paper, the grammar guided genetic programming (GGGP) and the particle swarm optimization (PSO) algorithm were used to optimize the structure and parameters of FNT, respectively.

#### 2.2.1. Grammar Guided Genetic Programming

Genetic programming is an evolutionary computational method proposed by Koza [19]. In terms of operators for population evolution, genetic programming algorithm replaces the binary string representation of genetic algorithms and operates on more intuitive population individuals. The genetic algorithm mainly utilizes the idea of superiority and inferiority in biology. It calculates a predetermined fitness function using a hierarchical structure with a more flexible expression form. After that, it simulates the process of biological genetic evolution and searches for the best one in the solution space through genetic operations such as selection, crossover, and mutation.

GGGP overcomes the drawback that the set of functions and terminal instructions must be of the same type in standard genetic programming algorithms and avoids the problem of generating invalid individuals in the process of crossover or mutation. When using GGGP to generate chromosomes of individuals in a population, the context-independent grammar *G* is defined by a quadruplet {*N*, *T*, *P*, Σ}, where *N* is a set of nonterminal symbols, *T* is a set of terminal symbols, *P* is a set of generation rules, and Σ is a starting symbol. If *x* ∈ *N* and *y* ∈ *N* ∪ *T* are defined, the grammar rule is denoted as *x*⟶*y*. The following is the algorithmic flow for generating GGGP:

1. Randomly generate the initial population according to the grammar model individual generation method
2. Calculate the fitness of individual in the population to evaluate each individual currently
3. Generate the next-generation population by genetic operations (including selection, crossover, and mutation), and evaluate all individual trees according to predefined criteria and individual fitness
4. If an individual optimal solution appears or the termination condition is met, the program ends; otherwise go to step (2) and continue the evolution

In the population generated by the optimization process, the quality of the individual is judged by the value of the fitness function Fit(*i*). The smaller the value, the smaller the error between the actual and expected output of the algorithm. In this paper, we use the standard variance as the fitness function:

(2) $$\begin{array}{c} \text{Fit}\left(i\right)=\sqrt{\frac{1}{N}\sum {\left(y_{1}^{i}-y_{2}^{i}\right)}^{2}}, \end{array}$$

where *N* represents the total number of individuals. *y*_1 _^*i*^ and *y*_2_^*i*^ represent the actual value of the *i*-th individual and the output value of the algorithm, respectively. The smaller the value of Fit(*i*), the better the individuals we get.

#### 2.2.2. Particle Swarm Optimization

The particle swarm optimization (PSO) algorithm proposed by Kennedy was a population-based heuristic search algorithm [20]. PSO represents the solution space of a problem as the search space of particles and the solution of a problem as the position of particles. The velocity of the particles in the space determines the speed and direction of the particles' motion [21]. The fitness value of each particle can be obtained by calculating the objective function. In this way, the problem of finding the optimal solution is transformed into the problem of finding the optimal position of the particle in the search space.

**Figure 1:**
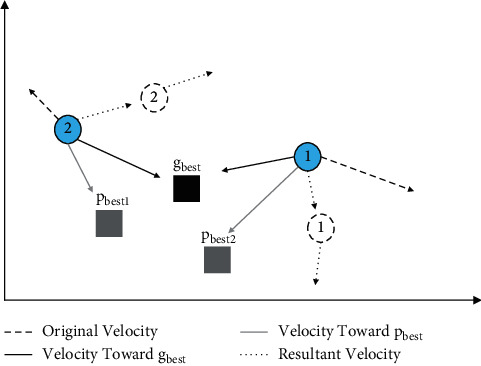
Schematic diagram of the basic principle of PSO, two moving particles 1 and 2 exist in two-dimensional space. Under the joint action of *p*_best_ and *g*_best_, both particles deviate from the original direction of motion and turn to the direction of the best position. In this way the example gets rid of the original local optimal position and gradually converges to the global optimal position.

When using the particle swarm optimization algorithm, the swarm of particles is first randomly initialized in the given solution space, and each particle then has an initial position and an initial velocity [22]. Iteration is performed according to the set rules: each evolution particle updates its position and speed in the solution space according to *p*_best_ and *g*_best_, where *p*_best_ is the optimal solution obtained by a single particle in the evolution process and *g*_best_ is the optimal solution obtained by all particles in the evolution process. After obtaining these two optimal solutions, particle *i* can update its velocity and position according to formulas (3) and (4):

(3) $$\begin{array}{c} v_{i}=\omega \times v_{i}+c_{1}\times r_{1}\times \left(p_{{\text{best}}_{i}}-x_{i}\right)+c_{2}\times r_{2}\times \left(g_{\text{best}}-x_{i}\right), \end{array}$$

(4) $$\begin{array}{c} x_{i}=x_{i}+v_{i}, \end{array}$$

where *v*_*i*_ is the velocity of particle *i*, *x*_*i*_ is the position of particle *i*, *c*_1_  and *c*_2_ are learning factors, *r*_1_=rand(0,1), *r*_2_=rand(0,1). *ω* is the inertia factor. The particle constantly adjusts its position according to the velocity but cannot exceed the maximum velocity *V*_max_, which will be restricted to *V*_max_ when *v*_*i*_ exceeds *V*_max_.  Figure 1 further illustrates the PSO optimization process.

The particle swarm optimization algorithm mainly contains six basic steps: ① initialize relevant parameters; ② calculate the fitness value of particles; ③ find out the current optimal solution of each particle and set it as *p*_best_; ④ find out the current optimal solution of the whole particle swarm and set it as *g*_best_; ⑤ update the velocity and position of the particle according to formulas (3) and (4); ⑥ go to step ② until satisfactory results are achieved or the termination conditions are met.

#### 2.2.3. Flexible Neural Tree

**Figure 2:**
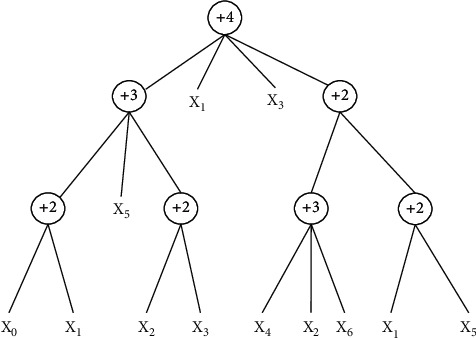
A flexible neural tree model.

Compared with other forms of deep neural networks, the FNT model can automatically optimize its own structure during training, without the need for the user to set it up in advance. During the structure optimization process, FNT allows cross-layer linkage between input vectors and individual nodes, which can produce some sparse networks with good performance relatively easily. It is difficult to achieve this when setting the network structure based on human experience. [23] In addition, FNT can automatically select key features by assigning different weights to input vectors during the training process, which greatly improves the classification ability of the model.  Figure 2 is a flexible neural tree model.

**Figure 3:**
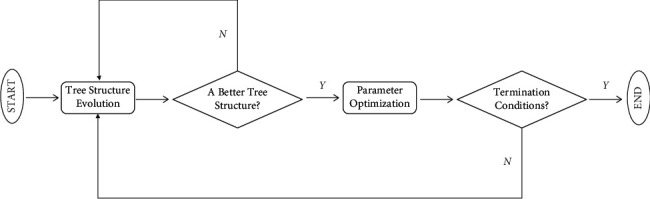
FNT algorithm flowchart.

To construct a flexible neural tree as shown in Figure 2, we first defined the model as *S*=*F* ∪ *T*={+2, +3, +5} ∪ {*x*_0_, *x*_1_,…,*x*_6_}, where *F*={+2, +3, +4} is the nonleaf node (function set), and FNT can produce more diverse tree network structures by changing the function set [24]. *T*={*x*_0_, *x*_1_,…,*x*_6_} is the leaf node (terminal instruction set). The algorithm flow of FNT is shown in Figure 3.

Initialize the relevant data; randomly assign the network parameters and generate the initial tree structure; then optimize the tree structure by grammar guided genetic programming and repeat this step until a better tree structure is found. Use particle swarm optimization to optimize the network parameters of the current optimal tree structure and repeat this step until the optimal parameter value is found or the number of iterations exceeds the set value. Finally the current generated network structure and parameters are judged according to the termination condition whether they can meet the requirements, and if not, the network is regenerated by going back to the tree structure optimization step until the termination condition is met. In calculating the output of FNT, we use *σ*(*x*)=1/1+exp(−*x*) as the activation function of nonleaf nodes [25]. The algorithm rules of the FNT model are shown in Table 2.

### 2.3. Ensemble Method

**Figure 4:**
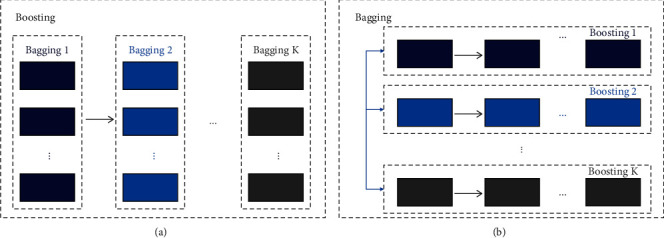
(a) Boosting ensembled with multiple bagging algorithms; (b) bagging ensembled with multiple boosting algorithms.

Ensemble learning combines multiple learners with different ensemble strategies to train them to perform the same task. Ensemble learning models usually have better generalization performance than individual learners [27]. There are mainly two common ways to integrate learners: one is to generate base learners without dependencies on each other in parallel by bagging ensemble strategies [28]. The representative algorithm is Random Forest (RF) [29]. The ensemble strategy of bagging can effectively improve the accuracy of the algorithm; the other is through boosting ensemble strategy [30], which can serially generate base learners with strong dependence between each other. And the representative algorithms are AdaBoost and Gradient Boosting Decision Tree (GBDT) [31]. Boosting ensemble strategy can reduce the risk of model overfitting and improve the generalization ability of the model.  Figure 4 gave a block diagram combining the two ensemble approaches of bagging and boosting.

### 2.4. The Cascade Flexible Neural Forest Model

Cancer subtype gene expression data is a continuous type of data. Different gene expression information had strong correlation, which means that it is better not to discretize them when classifying cancer subtypes on gene expression data. This will lead to various classifiers (such as decision trees and random forests) showing poor performance in the classification of cancer subtypes [32]. FNT is a new form of neural network, which has many advantages that traditional deep neural network models do not have (for example, the structure and parameters of the model can be automatically optimized, feature selection can be performed automatically, etc.) [23, 33].

**Figure 5:**
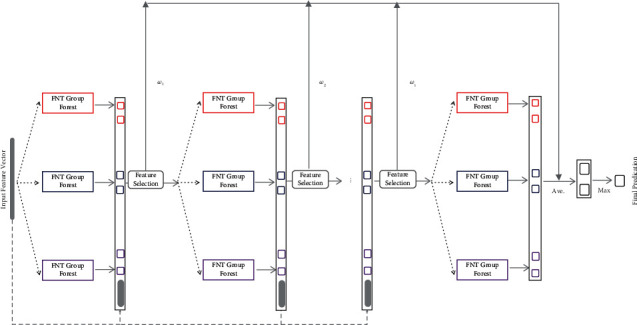
A Cascade Flexible Neural Forest model. We generate three forests by different grammars. The red forest used the function set {+2, +3, +4}; the blue forest used the function set {+2, +3, +5}; the purple forest used the function set {+2, +4, +5}.

However the single output characteristic determined that it cannot be directly used to deal with multiclassification. Therefore, it needs to be further optimized. Secondly, gene expression data is expensive to obtain and the number of samples available is small. But the good performance of deep neural networks often depends on a large number of training samples. Too few samples will lead the model to suffer from poor performance caused by overfitting [34]. Therefore, it is necessary to use new methods to improve the ability of FNT to use samples and make the model suitable for processing small sample size datasets. In addition, in order to obtain better classification performance and enhance the generalization ability of the model, we need to do further deepening of FNT. The common deepening methods not only add many hyperparameters to the model, but also bring high calculation cost. So a more appropriate framework should be used to deepen the FNT. Based on this, this paper proposed the Cascade Flexible Neural Forest model (CFNForest) as shown in Figure 5.

**Figure 6:**
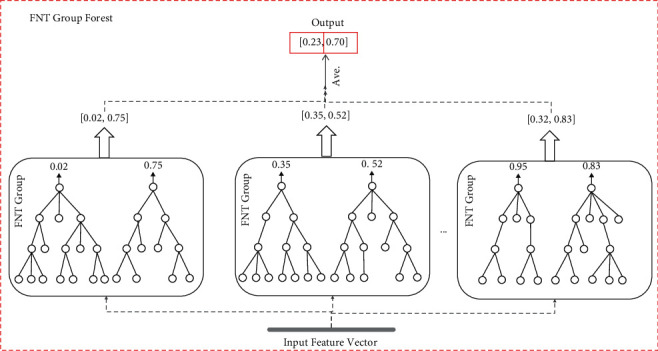
Structure of FNT Group Forest. Each forest has *K* FNT Groups. For the four-classification problem, there are 2 FNTs in each group. For all samples, FNT Group will produce a result such as {[0.02, 0.75] [0.35, 0.52] … [0.95, 0.83]}. At this time, the classification results of the whole FNT Group Forest for this sample were averaged, and the classification results of the whole FNT Group Forest for this sample were obtained as follows: [0.23, 0.70].

The FNT model has only one root node as its output, which determined that it can only do binary classification problems, while cancer tends to have multiple subtypes. In order to make the FNT model work well in the field of cancer subtype classification, the *M*-ary method was exploited to transform a multiclassification problem into several binary problems. Then the FNT Group was generated according to the number of generated binary problems, which means that there are log_2_  *M* FNTs in a group (*M* is the arity). We took a FNT Group as a whole and used the bagging ensemble strategy to form a FNT Group Forest and put *K* FNT Groups on each node. The number of *K* can be set according to the demand (increasing the number of FNT Groups in the forest is conducive to improving the classification performance of the forest, but at the same time, it will increase the overall computing cost; lowering the value of *K* can improve the operating efficiency of the model but may affect the overall classification accuracy of the forest).  Figure 6 shows the structure and algorithm inside the FNT Group Forest.

In order to improve the classification accuracy of cancer subtypes by deepening FNT, The boosting strategy was adopted to ensemble multiple powerful parallel FNT Group Forests. The advantage of this framework is that it cannot only make the model take the advantage of ensemble learning and improve its generalization ability, but also deepen the FNT without introducing new hyperparameters. To build a good ensemble framework, the individual learners should be diverse [35]. Therefore, we use three different function sets of FNT Group Forest in our model.

After getting the output of the first layer of the forest, we used the classification results of the forest as enhancement samples. We combined them with the original features and passed them on as new inputs to the next layer. Through this internal feature transformation, we can effectively improve the model performance and enhance the classification accuracy. In this way, each layer of the forest gets log_2_  *M∗*3 enhancement samples. As the number of layers increased, the entire forest can use more and more enhancement samples in training. This will make the model still have outstanding performance when dealing with small sample size datasets. With the depth of the model increasing, this advantage will become more and more obvious.

In order to improve the operational efficiency of CFNForest and reduce the computational complexity of the model, we introduced a sample selection mechanism after each layer of the forest: setting a prediction confidence to divide the samples into two parts. One is the sample that has reached a high classification accuracy, which is called sample set *Y*. The other one is the sample that does not meet the confidence requirement, which is called sample set *X*. For example, the prediction confidence interval is given as [0 ~ 0.2] ∪ [0.8 ~ 1]. There are 5 samples processed in the first layer, and the classification results are {[0.07,0.03] [0.35,0.44] [0.52,0.67] [0.83,0.12] [0.95,0.14]}. We can then split this sample set into two parts: the two samples with output results of [0.07, 0.03] and [0.95, 0.14] are within the confidence interval we set, so these two samples belong to sample set *Y*. The other three samples that do not meet the confidence requirement belong to sample set *X*.

For each sample in *Y*, we have enough certainty about which cancer subtype they belong to, and we do not need to send them to the next level for reclassification. As for the sample set *X*, we believe that the current forest structure cannot classify them effectively. Therefore, it is necessary to continue to increase the depth of the model and optimize the overall structure. The main purpose of setting output optimization mechanism is to solve the problem of excessive computational complexity caused by meaningless computation. As the number of forest layers increases, the number of samples that meet the confidence level requirement will increase layer by layer. At the same time, fewer and fewer samples will need to be sent to the next level for further classification. In this experiment, the training set is divided into two parts, one for training and the other for validation. When a new layer is added, the validation set will validate the whole forest. When adding new forests does not increase the accuracy anymore or the accuracy increase is not significant, the number of layers in the model will stop increasing. In this way, the model can automatically determine its number of layers.

In order to avoid classification errors caused by the difference between the classifications results of one layer of the forest and other layers for the same sample, when calculating the final output of the model, we let the classification results of each layer participate in the calculation of the final output with different weights. The final result *y*_*f*_ was calculated as follows:

(5) $$\begin{array}{c} y_{f}=\sum_{i=1}^{N}\omega_{i}*y_{i}\left(\omega_{i}=i/1+2+\cdots +i\right), \end{array}$$

where *N* is the number of layers, *y*_*i*_ is the output of the *i*-th layer forest, and *ω*_*i*_ is the weight of the classification result of the *i*-th layer forest to the final output.

## 3. Results

### 3.1. Comparison of CFNForest with Other Classifiers on BRCA Datasets

**Figure 7:**
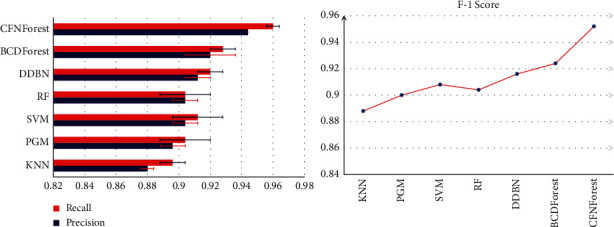
Overall performance comparison of multiple classifiers on BRCA datasets. The average precision, recall, and F-1 score were evaluated.

We tested the classification performance of the model on CFNForest for cancer subtypes. In order to demonstrate the superiority of CFNForest in classification performance, we compared it with k-nearest neighbor (KNN), the probabilistic graphical model (PGM) [11], support vector machine (SVM), random forest (RF), discriminative deep belief network (DDBN) [13], and boosting cascade deep forest (BCDForest) [12], respectively. The gene information obtained after feature processing is used as the input to each classifier. For all input samples, we used a fivefold cross-validation method to evaluate the overall performance of each classifier. The full dataset was divided into 5 subdatasets proportionally following class cardinalities; each of the 5 parts was taken in turn as testing data (and the remaining as training data) in each of the 5 runs of a cross-validation experiment. For each classifier, the classification results were compared in terms of average precision, recall, and F-1 score (as shown in Figure 7).

When classifying cancer subtypes of BRCA, the classification accuracy of CFNForest reached 94.4%. The classification performance was superior to other classifiers. In Figure 7, we compared the deviation values of classification accuracy and recall in 10 experiments. Experimental results demonstrated that our proposed CFNForest not only achieved higher classification accuracy, but also had more stable classification results compared with other classifiers, which indicated that this model has good robustness in classification performance.

### 3.2. Comparison of CFNForest with Other Classifiers on GBM Datasets

**Figure 8:**
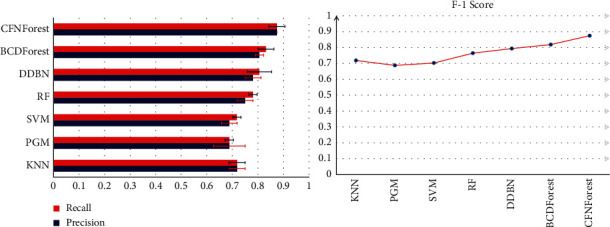
Overall performance comparison of multiple classifiers on GBM datasets. The average precision, recall, and F-1 score were evaluated.

We proceeded to test the classification performance of the proposed model on the GBM dataset for cancer subtypes. It was compared with KNN, PGM, SVM, RF, DDBN, and BCDForest. To ensure the accuracy of the experimental results, the fivefold cross-validation was used to evaluate the overall performance of each classifier. The evaluation results of each classifier in terms of average precision, recall, and F-1 score are shown in Figure 8.

As shown in Figure 8, the classification accuracy of CFNForest is significantly better than other classification models. Compared with KNN, PGM, SVM, RF, DDBN, and BCDForest, CFNForest still showed better classification performance on GBM datasets. At the same time, we can notice that the classification accuracy of all classifiers was much lower than the results obtained on the BRCA dataset. We think this is because the sample size of the training set is too small. The emergence of such a situation also reaffirms what we have mentioned before: with the limited number of available training samples, how to build a better model to achieve accurate classification for each sample is still a problem that needs further consideration.

### 3.3. Comparison of CFNForest with Other Classifiers on LUNG Datasets

**Figure 9:**
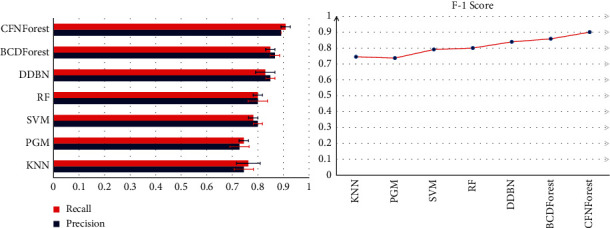
Overall performance comparison of multiple classifiers on LUNG datasets. The average precision, recall, and F-1 score were evaluated.

Finally, we tested the performance of the proposed model for the classification of these cancer subtypes on the LUNG dataset. We let it be compared with KNN, PGM, SVM, RF, DDBN, and BCDForest. The gene information obtained after feature processing is used as the input of each classifier. For all input feature vectors, this paper used the fivefold cross-validation method to evaluate the overall performance of each classifier. The classification results of each classifier were compared in terms of average precision, recall, and F-1 score, and the results are shown in Figure 9.

CFNForest achieved a classification accuracy of 90.9% on the LUNG dataset, which is better than other classification models. Compared with KNN, PGM, SVM, RF, DDBN, and BCDForest, the proposed model still showed better classification performance on this dataset. Experiments on the standard deviation of classification accuracy showed that the experimental results obtained by CFNForest are more stable than other classification models.

## 4. Discussion

The correlation between gene expression information is very strong, while decision trees as well as random forests often lose information in the process of discrete input features, which in turn destroy the correlation between genes. Therefore, this type of classifier tended to exhibit poor performance in cancer subtype classification problems. Deep neural networks, as a multilayer parametric model trained by backpropagation, are well suited to process continuous data such as genetic information because of their nonlinear and differentiable characteristics. However, the structure of traditional deep neural network models was generally designed based on the researcher's experience. There are almost infinite ways to combine the model structure. Therefore, it is necessary to use a more appropriate neural network model to process gene expression data to achieve accurate classification of cancer subtypes.

Gene expression data are costly to acquire and the number of samples available is small. However, the training process of deep neural networks usually required a large amount of training data. Otherwise, the model performance is not only poor, but also easy to fall into the risk of overfitting. Therefore, new optimization strategies need to be proposed to improve the way deep neural networks use training samples and make them suitable for processing small sample size datasets.

In this paper, a novel deep neural network ensemble model was proposed. Based on the traditional flexible neural tree model, it was extended to FNT Group Forest using bagging ensemble strategy, which can not only generate good neural network structure and parameters adaptively, but also overcome the problem that FNT cannot be used for multiple classification tasks. The FNT model was deepened with a cascade framework, and the model performance was improved by feature transformation within the model. Immediately after that, the robustness of the algorithm is ensured by setting different weights for each layer as well as introducing the feature optimization mechanism to the forest, which improves the efficiency of the model operation. Finally, FNT Group Forest with different functional sets was introduced to enrich the diversity of model structure, making it more suitable for processing small sample size datasets.

In the current medical diagnosis, the diagnosis of most cancer patients is based on the medical knowledge and experience of medical practitioners. And cancer has certain incubation period, the early lesions phenomenon is not very significant, and once a medical worker is on a business trip, the patient's condition is often easy to miss the best treatment period. Therefore, how to effectively diagnose cancer and correctly identify its subtypes is extremely important for the medical field and the maintenance of human life.

The gene expression of cancer tissue cells is different from that of normal tissue cells, which provides an effective basis for elucidating the pathogenesis and developmental characteristics of cancer from the perspective of genes [2]. The analysis of gene expression data provides guidance and decision-making for early diagnosis of cancer, which has positive and far-reaching significance for the correct and timely diagnosis of cancer. Of course, the study of cancer on gene expression data only provides clinicians with an alternative approach, and the personalized treatment of cancer on other molecular biological characteristics [36] will also be a direction we will focus on in future research.

## 5. Conclusions

As one of the major causes of human death, cancer has the characteristics of multiple types and complex pathogenesis. The pathological changes in cancer tend to occur at any time. Objective and accurate classification of cancer subtypes enables doctors to correctly understand the pathogenesis and primary location of cancer, which is of great significance to the study of cancer genesis. The comprehensiveness of gene expression data and the high correlation between genes and cancers make it a natural advantage to conduct classification studies of cancer subtypes on gene expression data. Gene expression data is essentially a high-dimensional, small sample size, and highly redundant data. There are great difficulties in feature processing and model design on these data using traditional classifiers. In this paper, a new cancer subtype classification model, CFNForest, was proposed, which is capable of hierarchical processing of gene features without discrete input features compared with deep models built on nondifferentiable models. Compared to traditional deep neural networks, the proposed model can automatically optimize the internal structure and parameters during the training process. CFNForest uses a framework that integrates multiple bagging algorithms using a boosting strategy. On the basis of the advantages of ensemble learning, the overall performance of the model is improved by feature transformation between layers, which in turn enables it to obtain high classification accuracy on small sample size datasets. Experimental results show that CFNForest consistently outperforms the state-of-the-art methods in classifying breast invasive carcinoma, glioblastoma multiforme, and lung cancer by using RNA-seq gene expression data.

## Figures and Tables

**Table 1:** The detail of the three cancer types.

Dataset	Sample	Gene	Class
BRCA	514	4247	4
GBM	164	3398	4
LUNG	275	4596	3

**Table 2:** Algorithm rules of flexible neural tree [26].

(1) The output of the leaf node is the given input characteristic variable value
(2) The output of nonleaf node +*M* is *y*_non−leaf_=*σ*(∑_*j*=0_^*M*^*ω*_*j*_*I*_*j*_+*θ*), where, *I*_*j*_ is the input of the current node, *ω*_*j*_ is the corresponding weight and *θ* is the bias of nodes
(3) The output of each node is treated as input to the node at the upper level to which it is connected
(4) Calculate the value of the output vector from the bottom up, from the leaf node to the root node

## Data Availability

The gene expression data and miRNA expression data can be downloaded from The Cancer Genome Atlas website at https://www.cancer.gov/about-nci/organization/ccg/research/structural-genomics/tcga/. The specific BRCA, GBM, and LUNG datasets in our manuscript were available through https://github.com/VeblenChung/Cancer-Subtype-Classification-Data-Set.

## References

[1] Bersimbaev R., Bulgakova O., Aripova A., Kussainova A., Ilderbayev O. (2021). Role of microRNAs in lung carcinogenesis induced by asbestos. *Journal of Personalized Medicine*.

[2] Golub T. R., Slonim D. K., Tamayo P. (1999). Molecular classification of cancer: class discovery and class prediction by gene expression monitoring. *Science*.

[3] Wu J., Hicks C. (2021). Breast cancer type classification using machine learning. *Journal of Personalized Medicine*.

[4] Shen R., Olshen A. B., Ladanyi M. (2009). Integrative clustering of multiple genomic data types using a joint latent variable model with application to breast and lung cancer subtype analysis. *Bioinformatics*.

[5] Khan J., Wei J. S., Ringnér M. (2001). Classification and diagnostic prediction of cancers using gene expression profiling and artificial neural networks. *Nature Medicine*.

[6] Xia C.-Q., Han K., Qi Y., Zhang Y., Yu D.-J. (2018). A self-training subspace clustering algorithm under low-rank representation for cancer classification on gene expression data. *IEEE/ACM Transactions on Computational Biology and Bioinformatics*.

[7] Chu Y., Kaushik A. C., Wang X. (2021). DTI-CDF: a cascade deep forest model towards the prediction of drug-target interactions based on hybrid features. *Briefings in Bioinformatics*.

[8] Alfred G. (1991). Knudson, overview: genes that predispose to cancer. *Mutation Research: Fundamental and Molecular Mechanisms of Mutagenesis*.

[9] Fields C., Adams M. D., White O., Venter J. C. (1994). How many genes in the human genome?. *Nature Genetics*.

[10] Fakoor R., Ladhak F., Nazi A., Huber M. Using deep learning to enhance cancer diagnosis and classification.

[11] Khademi M., Nedialkov N. S. Probabilistic graphical models and deep belief networks for prognosis of breast cancer.

[12] Guo Y., Liu S., Li Z., Shang X. (2018). BCDForest: a boosting cascade deep forest model towards the classification of cancer subtypes based on gene expression data. *BMC Bioinformatics*.

[13] Karabulut E. M., Ibrikci T. (2017). Discriminative deep belief networks for microarray-based cancer classification. *Biomedical Research*.

[14] Xiao Y., Wu J., Lin Z., Zhao X. (2018). A deep learning-based multi-model ensemble method for cancer prediction. *Computer Methods and Programs in Biomedicine*.

[15] Weinstein J. N., Collisson E. A., Collisson E. A. (2013). The cancer genome atlas pan-cancer analysis project. *Nature Genetics*.

[16] Chen Y., Yang B., Dong J., Abraham A. (2005). Time-series forecasting using flexible neural tree model. *Information Sciences*.

[17] Chen Y., Yang B., Abraham A. (2007). Flexible neural trees ensemble for stock index modeling. *Neurocomputing*.

[18] Chen Y., Yang B., Meng Q. (2012). Small-time scale network traffic prediction based on flexible neural tree. *Applied Soft Computing*.

[19] Koza J. R. (1992). *Genetic Programming: On the Programming of Computers by Means of Natural Selection*.

[20] Kennedy J., Eberhart R. Particle swarm optimization.

[21] Bansal J. C. (2019). Particle swarm optimization. *Evolutionary and Swarm Intelligence Algorithms*.

[22] Sengupta S., Basak S., Peters R. A. (2019). Particle swarm optimization: a survey of historical and recent developments with hybridization perspectives. *Machine Learning and Knowledge Extraction*.

[23] Chen Y., Abraham A., Yang B. (2006). Feature selection and classification using flexible neural tree. *Neurocomputing*.

[24] Wu P., Wang D. (2019). Classification of a DNA microarray for diagnosing cancer using a complex network based method. *IEEE/ACM Transactions on Computational Biology and Bioinformatics*.

[25] Wu P., Orchard J. Using flexible neural trees to seed backpropagation.

[26] Xu J., Wu P., Chen Y., Meng Q., Dawood H., Khan M. M. (2019). A novel deep flexible neural forest model for classification of cancer subtypes based on gene expression data. *IEEE Access*.

[27] Zhou Z.-H. (2009). Ensemble learning. *Encyclopedia of Biometrics*.

[28] Hong H., Liu J., Zhu A. X. (2020). Modeling landslide susceptibility using logit boost alternating decision trees and forest by penalizing attributes with the bagging ensemble. *The Science of the Total Environment*.

[29] Pal M. (2005). Random forest classifier for remote sensing classification. *International Journal of Remote Sensing*.

[30] Drucker H., Cortes C., Jackel L. D., LeCun Y., Vapnik V. (1994). Boosting and other ensemble methods. *Neural Computation*.

[31] Rao H., Shi X., Rodrigue A. K. (2019). Feature selection based on artificial bee colony and gradient boosting decision tree. *Applied Soft Computing*.

[32] Pang M., Ting K., Zhao P., Zhou Z. Improving deep forest by confidence screening.

[33] Xu J., Wu P., Chen Y., Meng Q., Dawood H., Dawood H. (2019). A hierarchical ensemble deep flexible neural forest framework for cancer subtype classification by integrating multi-omics data. *BMC Bioinformatics*.

[34] Hinton G. E., Salakhutdinov R. R. (2006). Reducing the dimensionality of data with neural networks. *Science*.

[35] Suh Y. J., Jung J., Cho B.-J. (2020). Automated breast cancer detection in digital mammograms of various densities via deep learning. *Journal of Personalized Medicine*.

[36] Amoroso N., Pomarico D., Fanizzi A. (2021). A roadmap towards breast cancer therapies supported by explainable artificial intelligence. *Applied Sciences*.

